# Long-term outcome of vestibular function and hearing in children with congenital cytomegalovirus infection: a prospective cohort study

**DOI:** 10.1007/s00405-022-07816-7

**Published:** 2023-01-16

**Authors:** Eeva Kokkola, Riina Niemensivu, Maija Lappalainen, Maarit Palomäki, Tea Nieminen, Suresh Boppana, Harri Saxèn, Laura Puhakka

**Affiliations:** 1 New Children’s Hospital, University of Helsinki and Helsinki University Hospital, Helsinki, Finland; 2 Department of Otorhinolaryngology-Head and Neck Surgery, Hearing Clinic, Helsinki University Hospital, Helsinki, Finland; 3 HUS Diagnostic Center, HUSLAB, Clinical Microbiology, University of Helsinki and Helsinki University Hospital, Helsinki, Finland; 4 Department of Neuroradiology, HUS Medical Imaging Center, University of Helsinki and Helsinki University Hospital, Helsinki, Finland; 5 Department of Pediatrics, Heersink School of Medicine, University of Alabama at Birmingham, Birmingham, AL USA; 6 Department of Microbiology, Heersink School of Medicine, University of Alabama at Birmingham, Birmingham, AL USA

**Keywords:** Congenital cytomegalovirus infection, Sensorineural hearing loss, The video head impulse test, Vestibular dysfunction

## Abstract

**Purpose:**

Congenital cytomegalovirus infection (cCMV) is the most frequent nonhereditary cause for sensorineural hearing loss (SNHL) in children. Data on vestibular function in children with cCMV are, however, scarce, although some evidence for cCMV-associated vestibular dysfunction exists. In this prospective cohort study, we evaluated long-term vestibular function and hearing outcomes in a cohort of children with cCMV.

**Methods:**

Participants were 6–7-year-old children with cCMV from a large population-based screening study. Controls were age and gender matched healthy children, who were CMV-negative at birth. Hearing was examined with pure tone audiometry. Definition of hearing loss was pure-tone average > 20 dB. Vestibular function was assessed using the video head impulse test that provides a measure of semicircular canal function. Definition of vestibular dysfunction was lateral semicircular canal gain < 0.75.

**Results:**

Vestibular dysfunction occurred in 7/36 (19.4%) of children with cCMV and in 1/31 (3.2%) of controls (*p* = 0.060). SNHL was recorded in 4/38 (10.5%) of children with cCMV and in 0/33 of controls (*p* = 0.118). Hearing loss was unilateral in all cases. In cCMV group, the two children with bilateral vestibular dysfunction also had SNHL, whereas those with unilateral vestibular dysfunction (*n* = 5) had normal hearing.

**Conclusions:**

In this cohort of children with cCMV identified using newborn screening, vestibular dysfunction was more common than SNHL at 6 years of age. Vestibular dysfunction occurred both in children with and without SNHL. Based on these data, inclusion of vestibular tests in follow-up protocol of cCMV should be considered.

## Introduction

Congenital cytomegalovirus infection (cCMV) is the most common congenital infection and the main cause of non-genetic hearing loss in children [1]. Cytomegalovirus infects fetal inner ear structures [2, 3] that contain sensory receptors for hearing and balance. In addition to hearing loss, vestibular and balance dysfunction have been reported [4, 5], but data from population-based screening studies are lacking.

Only about 10% of newborns with cCMV have clinical findings (symptomatic infection) including intrauterine growth restriction, microcephaly, central nervous system abnormalities, chorioretinitis, petechiae, and jaundice with direct hyperbilirubinemia [6]. Thus, the majority of newborns with cCMV are asymptomatic and not recognized as CMV infected [6]. Of the symptomatic children with cCMV, 40–58% will develop long-term sequelae, such as cognitive, motor, hearing, and visual impairments, whereas only about 13.5% of asymptomatic children do [6, 7]. Sensorineural hearing loss (SNHL) is the most frequent sequela of cCMV [7] occurring in about 30–40% of symptomatic and 10% of asymptomatic children [8]. Emerging evidence suggests [5] that a significant proportion (~ 40%) of children with cCMV experience vestibular dysfunction.

Vestibular dysfunction and balance disorders have been reported in children with cCMV [4, 5, 9–12], but evidence from prospective population-based cohorts is scarce. It is crucial to study the long-term burden of vestibular dysfunction in cCMV, because vestibular dysfunction may result in functional deficits in children with cCMV [5]. The characteristics of hearing loss in cCMV are variable; it can be early or late-onset, fluctuating, or progressive [13]. Of the children with both symptomatic and asymptomatic cCMV and SNHL, the majority suffer from severe to profound hearing loss [13]. SNHL is more frequently bilateral in symptomatic children than those with asymptomatic cCMV [13].

Children with vestibular dysfunction may suffer from vertigo, falls, delayed motor development, and reading difficulties [14], whereas hearing loss may result in poorer cognitive and language skills, social life, education, and quality of life [15]. A 6-year hearing follow-up is recommended for children with cCMV [13]. Most children with asymptomatic cCMV are not recognized, and therefore, sufficient long-term outcome data are not available from this group [16]. Even in children who were identified as having cCMV, monitoring of vestibular function is not routinely included in the follow-up. In this prospective cohort study, we report vestibular and hearing outcomes in 6–7-year-old children with cCMV, identified in a population-based neonatal cCMV screening.

## Methods

The study was conducted between September 2018 and December 2021 in Helsinki University Hospital, Finland. Study protocol was approved by the Ethics Committee for Women, Children, and Psychiatry in the Hospital District of Helsinki and Uusimaa. Informed consent was collected from parents or legal guardians of participants before enrollment.

### Participant characteristics

Study population consisted of 6–7-year-old children with cCMV (*n* = 40), identified in a previously reported prospective population-based cCMV screening [17], and healthy CMV-negative controls (*n* = 54) matched by sex, gestational age, date of birth, and neonatal ward. The CMV screening with saliva real-time PCR [18] was conducted for 19,868 Finnish newborns between 2012 and 2015. None of the children with cCMV received antiviral medication. Infants with cCMV were enrolled in a prospective follow-up study that included hearing testing at 3, 9–12, 18 months, and at 3 years for children with cCMV [17, 19]. The control group attended the same follow-up except hearing testing at 9–12 months and 3 years.

At 6 year age, we performed MRI of inner ear to exclude inner ear pathologies. Imaging was performed with 3 Tesla MRI system (Philips Ingenia 3.0T). Image sequence was 3D Drive. No anesthesia, sedative medication, or contrast agent was used during imaging. One experienced pediatric neuroradiologist reviewed the images.

### Vestibular testing

Vestibular function was studied using the video head impulse test (vHIT) which measures vestibular function in individual ears and is based on the detection of abnormalities in vestibulo-ocular reflex. In vHIT, participant fixes eyes to a still target, and eye movements are filmed during accelerated and decelerated head movements. vHIT reports gain, the ratio between eye and head movements. Different head positions allow assessing function of the three semicircular canal pairs one at a time. Compared to vertical semicircular canals, testing of lateral semicircular canals´ (LSCCs´) gain tends to be more reliable to complete in children [20, 21].

One experienced physiotherapist performed vHIT for all participants with the same device (ICS Impulse, Otometrics, Denmark). The gains of LSCCs of right and left ears were examined and a gain < 0.75 was considered abnormal. Participants with an abnormal gain in one or both ears were considered to have vestibular dysfunction. In addition to decreased gain, corrective saccades can be seen in vestibular dysfunction. Furthermore, to assess asymmetry, the difference between the gains of LCSSs of right and left ears was determined, and > 0.2 was considered abnormal.

### Hearing testing

Hearing was assessed using pure-tone audiometry (Interacoustics AC 40). An expert pediatric audiologist performed hearing testing in the same soundproof room that was acoustically suited for the examination. Children used calibrated headphones (Radioear 3045) and were asked to respond to pure tone stimuli. Hearing was assessed for each ear individually, and masking was used to prevent cross hearing by bone conduction from contralateral ear. Hearing thresholds were examined over frequencies of 125 Hz–8 kHz and the pure tone average (PTA) was assessed for each individual ear. PTA ≤ 20 dB was considered normal. Children with PTA > 20 dB in one or both ears were considered to have a hearing loss. EU classification was used to determine the degree of hearing loss in the worse ear. In case of hearing loss, bone thresholds were measured to determine the type of hearing loss (sensorineural, conductive, or mixed). In this study, we reported the cases with SNHL, including subjects with mixed hearing loss.

An experienced pediatric otolaryngologist examined all children with hearing loss to exclude middle ear pathologies, such as otitis media. We reviewed patient records to determine the onset of hearing loss. It was considered early onset in those with apparent hearing loss at the first follow-up visit at 3 months of age, and late-onset in those diagnosed with SNHL after that.

### Statistical methods

We used IBM SPSS Statistics 25 software for statistical analysis. Results of quantitative data were reported using mean ± SD, and results of qualitative data were reported as frequencies and percentages. To test associations between groups, Mann–Whitney *U* test was used for continuous variables and Fisher’s Exact test for categorical variables. A *p* value < 0.05 was considered statistically significant.

## Results

### Participant characteristics

Of the 40 children with cCMV, 38 (95.0%) participated in hearing testing, and 36 (90%) in vestibular testing. Of the 38 children with cCMV, 4 (10.5%) had symptomatic and 34 (89.5%) asymptomatic cCMV. Of the 4 children with symptomatic cCMV at birth, 3 had abnormal neuroimaging findings (cerebral calcifications on cranial ultrasound at 3 months of age) and one child had microcephaly. Of the 54 controls, 33 (61.1%) participated in hearing testing, and 31 (57.4%) in vestibular testing.

The mean age was 6.41 ± 0.39 years in cCMV group and 6.19 ± 0.23 years in the control group. 24 of 39 (61.5%) with cCMV and 17/33 (51.5%) controls were females. All children were born at term.

At 6 years of age, MRI of inner ear was performed for 21 children with asymptomatic cCMV and 27 controls. All images were normal.

### Vestibular testing

Among children with cCMV and controls, 19.4% and 3.2% (*p* = 0.060) had vestibular dysfunction, respectively (Table 1). Vestibular dysfunction was diagnosed in 1/4 (25%) symptomatic and 6/32 (18.8%) children with asymptomatic cCMV. The vestibular dysfunction was more frequently unilateral (5/7, 71.4%) in children with cCMV (Table 1).

**Table 1:** Vestibular function in children with cCMV and controls

	Children with cCMV	Controls	*p* value*
Vestibular dysfunction^a^, *n* (%)	7/36 (19.4%)	1/31 (3.2%)	0.060
Laterality of vestibular dysfunction			
Unilateral, *n* (%)	5/7 (71.4%)	1/1 (100%)	–
Bilateral, *n* (%)	2/7 (28.6%)	–	–
Gain in right ear, mean ± SD (range)	0.88 ± 0.17 SD (0.00–1.13)	0.91 ± 0.06 SD (0.76–1.00)	0.537
Gain in left ear, mean ± SD (range)	0.84 ± 0.15 SD (0.14–1.08)	0.85 ± 0.06 SD (0.71–0.96)	0.753
Gain asymmetry, mean ± SD (range)	0.074 ± 0.059 SD (0.00–0.24)	0.089 ± 0.056 SD (0.01–0.28)	0.193
Abnormal gain asymmetry^b^, *n* (%)	1/36 (2.8%)	1/31 (3.2%)	1.000

*cCMV* congenital cytomegalovirus infection, *vHIT* the video head impulse test

^*^Significance level < 0.05. Mann–Whitney *U* test was used for continuous variables and chi-squared test for categorical variables

^a^Lateral semicircular canal gain < 0.75 in vHIT

^b^> 0.2 asymmetry between the gains of right and left ears was considered abnormal

### Hearing testing

Of the 38 children with cCMV, 4 (10.5%) had SNHL (one with mixed hearing loss) compared to 0/33 controls (*p* = 0.118) (Table 2). Among children with symptomatic and asymptomatic cCMV, 1/4 (25%) and 3/34 (8.82%) had SNHL, respectively. In all children with SNHL, hearing loss was unilateral (Table 2). The degree of hearing loss in the poorer hearing ear ranged from mild (21 ≤ dB HL < 39) to profound (≥ 94 dB HL).

**Table 2:** Hearing in children with cCMV and controls

	All children with cCMV	Symptomatic cCMV	Asymptomatic cCMV	Controls
SNHL, *n* (%)	4/38 (10.5%)^a^	1/4 (25%)	3/34 (8.82%)^a^	0/33 (0%)
Laterality of SNHL				
Unilateral, *n* (%)	4/4 (100%)	1/1 (100%)	3/3 (100%)	–
Bilateral, *n* (%)	–	–	–	–
Degree of HL in poorer hearing ear				
Mild, 21 ≤ dB HL < 39, *n* (%)	2/4 (50%)	–	2/3 (66.6%)	–
Moderate 40 ≤ dB HL < 69, *n* (%)	1/4 (25%)	1/1 (100%)	–	–
Severe 70 ≤ dB HL < 94, *n* (%)	–	–	–	–
Profound ≥ 94 dB HL, *n* (%)	1/4 (25%)	–	1/3 (33.3%)	–
Onset of HL				
Early, *n* (%)	3/4 (75%)	1/1 (100%)	2/3 (66.6%)	–
Late, *n* (%)	1/4 (25%)	–	1/3 (33.3%)	–

*cCMV* congenital cytomegalovirus infection, *HL* hearing loss, *SNHL* sensorineural hearing loss

^a^One participant had mixed hearing loss

Otologic examination was normal in 3 participants with SNHL, and 1 participant with mixed hearing loss had Rinne test negative on the side of the poorer hearing ear.

All children with SNHL and their families received counselling about unilateral hearing loss and its consequences. Hearing aid fitting in unilateral hearing loss was an individual decision based on difficulties in hearing, delay in speech development, problems in concentration, or parent´s opinion. At that time, our national treatment policy did not suggest cochlear implantations for children with unilateral SNHL. Thus, none of the children with SNHL received hearing rehabilitation with hearing aid or cochlear implantation.

Hearing loss was recorded also at the age of 3 months (early onset) in 3/4 of the children with cCMV and at the age of 6 years (late-onset) in one child (Table 2). In addition, 2 children with cCMV were diagnosed with hearing loss at 3 years of age [19], but had normal hearing at 6 years of age, and therefore, it is possible that they had a fluctuating hearing loss but also lack of compliance or middle ear condition (such as underpressure in middle ear) might have contributed to hearing results at 3 year age.

### Association between vestibular dysfunction and SNHL in children with cCMV

In children with cCMV, 2/4 (50%) with SNHL also had vestibular dysfunction compared to 5/32 (15.6%) with normal hearing. Table 3 shows vestibular function and hearing results in every child with cCMV that had vestibular dysfunction and/or SNHL. Both children with bilateral vestibular dysfunction also had SNHL.

**Table 3:** Results in children with cCMV and vestibular dysfunction or SNHL

Patient number, type of cCMV	Onset of SNHL	Pure tone audiometry results	Laterality and degree of SNHL	vHIT results	Laterality of vestibular dysfunction
1, symp^a^	Early	SNHL (65/6 dB HL^b^)	Unilateral moderate	Normal (0.90/0.81^c^)	–
2, asymp	Early	SNHL (15/25 dB HL)	Unilateral mild	Normal (0.97/0.95)	–
3, asymp	Late	MHL^d^ (33/9 dB HL)	Unilateral mild	VD (0.7/0.69)	Bilateral
4, asymp	Early	SNHL (6/ > 118 dB HL)	Unilateral profound	VD (0.0/0.14, corrective saccades)	Bilateral
5, asymp	–	CHL^e^ (31/13 dB HL)	–	VD (0.63/0.83)	Unilateral
6, asymp	–	Normal (15/15 dB HL)	–	VD (0.79/0.69)	Unilateral
7, asymp	–	Normal (4/9 dB HL)	–	VD (0.80/0.74)	Unilateral
8, asymp	–	Normal (8/10 dB HL)	–	VD (0.81/0.70)	Unilateral
9, symp^a^	–	Normal (4/3 dB HL)	–	VD (0.79/0.73)	Unilateral

*asymp* asymptomatic congenital cytomegalovirus infection, *cCMV* congenital cytomegalovirus infection, *CHL* conductive hearing loss, *dB* decibel, *HL* hearing loss *LSCC* lateral semicircular canal, *MHL* mixed hearing loss, *PTA* pure tone average, *SNHL* sensorineural hearing loss, *VD* vestibular dysfunction, *vHIT* the video head impulse test, *symp* symptomatic congenital cytomegalovirus infection

^a^Calcifications in cranial ultrasound at 3 months of age

^b^PTA (dx./sin.)

^c^LSCC gain (dx./sin.)

^d^Mixed hearing loss was considered to associate with cCMV

^e^Conductive hearing loss is not associated with cCMV

**Fig. 1:**
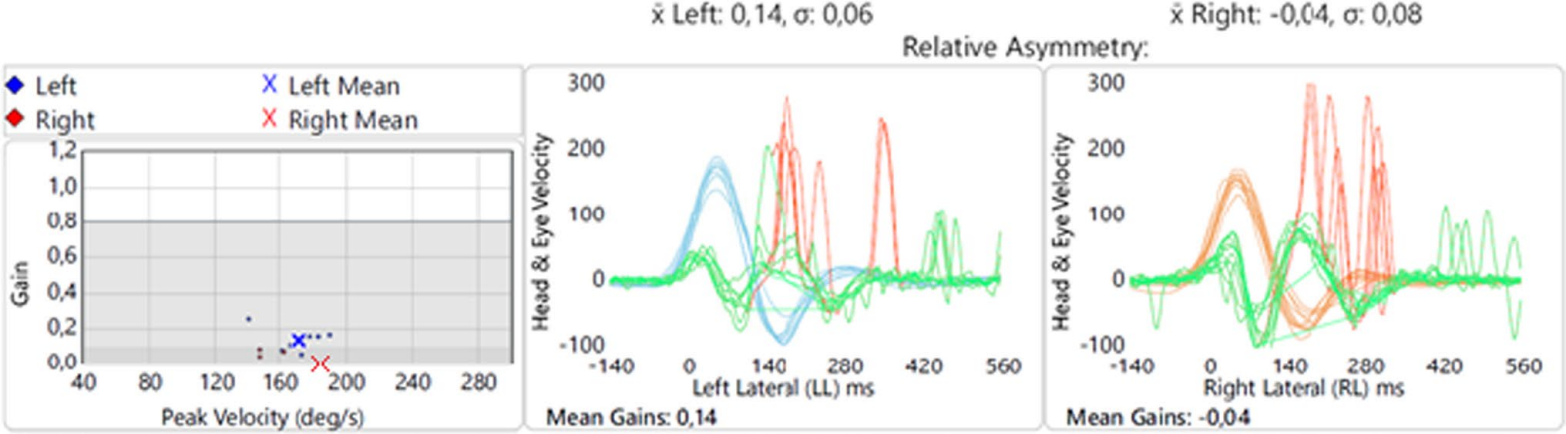
Results of vHIT testing in a child with profound unilateral hearing loss. *vHIT* the video head impulse test

Vestibular dysfunction and hearing loss were most severe in Patient 4, who had unilateral SNHL, apparent at 3 months of age. At the age of 6 years, when masking was used during PTA, left ear turned out to be deaf (Table 3). The vHIT gains were significantly low bilaterally, accompanied with corrective saccades (Table 3 and Fig. 1).

## Discussion

We studied long-term hearing and vestibular function outcomes in a cohort of 6–7-year-old children with cCMV, identified in a population-based neonatal CMV screening study [17]. Among children with cCMV, vestibular dysfunction (7/36, 19.4%) was more common than SNHL (4/38, 10.5%). Vestibular dysfunction and SNHL were more common in children with cCMV compared to controls.

Only a few previous studies have investigated vestibular dysfunction in cCMV [5, 11, 12, 22–24]. The incidence of vestibular dysfunction varied widely in these studies likely due to differences in factors, such as age of testing, follow-up duration, vestibular assessment tools, and the proportion of children with symptomatic and asymptomatic cCMV. In a retrospective study by Bernard et al*.* [22], 93.2% of children with cCMV had vestibular dysfunction, but all participants also suffered from severe hearing loss and needed evaluation for cochlear implantation. Hence, the study population was enriched for children with symptomatic cCMV. In the Pinninti et al*.* study, 17/38 (45%) asymptomatic children with cCMV, were diagnosed with vestibular dysfunction [12]. Although this cohort originated from a universal screening, only a small proportion (8.9%) of the total cohort of patients with cCMV were evaluated for vestibular function. However, the Pinninti et al*.* utilized a more comprehensive evaluation of vestibular function and balance. A larger prospective longitudinal study of 94 children with cCMV reported that 14% of the children had vestibular dysfunction [23]. The frequency of vestibular dysfunction in our study is similar to this study; however, the average age of the children followed was only 17 months [23], and thus, vestibular dysfunction that occurred later was not detected.

Even though vestibular dysfunction seems to be common in children with cCMV, the clinical relevance and the actual impact of this condition on the balance remain unclear. Vestibular dysfunction does not necessarily result in balance impairment, because children can compensate by vision and proprioception. We did not study balance outcome, but few previous studies have reported balance impairment in children with cCMV. Pinninti et al*.* [12] assessed balance and vestibular function in their cohort of asymptomatic children with cCMV, and showed that one-third to one-half of them had balance impairment. In a retrospective study, Karltorp et al*.* [4] reported that a high proportion (88%) of children with cCMV and severe hearing loss had balance impairment. These children learned walking without support significantly later compared to controls. More studies including both vestibular tests and balance follow-up are clearly required to evaluate the clinical relevance of vestibular dysfunction in cCMV.

The degree of hearing loss in our cCMV cohort was relatively mild. SNHL was diagnosed in 4/38 (10.5%) of children with cCMV, which is in line with a meta-analysis that reported that 12.6% of children with cCMV identified in universal screening had SNHL [13]. In our cohort, all children with cCMV and SNHL had unilateral hearing loss, and the degree of hearing loss was mild to moderate in all children except for one subject. Conversely, a systematic review reported a more severe degree as well as bilateral hearing deficits in both symptomatic and asymptomatic cCMV [13].

Due to the anatomic proximity of vestibular organ and cochlea, the association between vestibular dysfunction and SNHL is of interest. The findings of our study showed a higher risk for vestibular dysfunction in children with cCMV and SNHL compared to those with normal hearing. This confirms the findings of previous studies and demonstrate the significant burden of vestibular dysfunction in children with cCMV [11, 22, 23]. Although vestibular dysfunction was mainly unilateral in our study children with cCMV, the 2 children with bilateral deficits also had SNHL. This is consistent with a previous retrospective study [22], where 77% of children with cCMV with SNHL also had bilateral vestibular dysfunction. However, that study lacked a control group of normal-hearing children with cCMV. One of our participants with cCMV had unilateral profound SNHL and profound bilateral vestibular dysfunction. Bernard et al*.,* also reported a significant association between the severity of SNHL and vestibular dysfunction [22]. These data suggest that the subgroup of hearing-impaired children with cCMV are at increased risk for vestibular dysfunction.

Strengths of our study include that children with cCMV were identified on population-based screening in a prospective study and having a matched group of CMV-negative children as controls. Most children (38/40) from the original cohort, identified on newborn CMV screening, participated in this study. Most of the study participants had asymptomatic cCMV and would not have been recognized without newborn CMV screening. Another strength is that both vestibular and hearing testing was performed at 6–7 years of age and allowing us to obtain reliable data for individual ears.

The major study limitation was the small number of study children hindering the evaluation of the differences between symptomatic and asymptomatic cCMV. The differences in vestibular dysfunction and SNHL between children with cCMV and controls are not significantly different likely due to the small number of study subjects. Vestibular dysfunction was assessed using vHIT, because it was easy to perform, did not induce dizziness or nausea, and could be performed in a well-lit environment. However, vHIT does not test all aspects of the vestibular organ system and merely evaluates the function of semicircular canals and vestibular nerve. Therefore, the results of our study may have underestimated the true incidence of vestibular dysfunction. A more comprehensive testing protocol could have provided a more complete and accurate assessment of vestibular function and balance disorders [20].

## Conclusions

In this cohort of children with cCMV, vestibular dysfunction was more common than SNHL at 6–7 years of age. The findings of our study along with several recent reports suggest that clinicians should be aware of the possibility of vestibular and balance impairment in children with cCMV. Therefore, vestibular function and balance should be included in the follow-up of children with cCMV allowing early detection of children who might benefit from appropriate rehabilitation to ensure normal balance and motor development. In addition, there is an urgent need for future studies with larger number of children identified in newborn CMV screening undergoing systematic assessment of vestibular function and balance and to determine the disease burden and the effectiveness of timely intervention.

## Data Availability

The data that support the findings of this study are available on request from the corresponding author.

## Abbreviations

asymp: Asymptomatic congenital cytomegalovirus infection
cCMV: Congenital cytomegalovirus infection
CHL: Conductive hearing loss
HL: Hearing loss
LSCC: Lateral semicircular canal
MHL: Mixed hearing loss
PTA: Pure tone average
SNHL: Sensorineural hearing loss
Symp: Symptomatic congenital cytomegalovirus infection
VD: Vestibular dysfunction
vHIT: The video head impulse test

## References

[1] Nance WE, Lim BG, Dodson KM (2006). Importance of congenital cytomegalovirus infections as a cause for pre-lingual hearing loss. J Clin Virol.

[2] Teissier N, Delezoide AL, Mas AE, Khung-Savatovsky S, Bessières B, Nardelli J (2011). Inner ear lesions in congenital cytomegalovirus infection of human fetuses. Acta Neuropathol.

[3] Gabrielli L, Bonasoni MP, Santini D, Piccirilli G, Chiereghin A, Guerra B (2013). Human fetal inner ear involvement in congenital cytomegalovirus infection. Acta Neuropathol Commun.

[4] Karltorp E, Löfkvist U, Lewensohn-Fuchs I, Lindström K, Eriksson Westblad M, TeärFahnehjelm K (2014). Impaired balance and neurodevelopmental disabilities among children with congenital cytomegalovirus infection. Acta Paediatr.

[5] Shears A, Yan G, Mortimer H, Cross E, Sapuan S, Kadambari S (2022). Vestibular and balance dysfunction in children with congenital CMV: a systematic review. Arch Dis Child Fetal Neonatal Ed.

[6] Rawlinson WD, Boppana SB, Fowler KB, Kimberlin DW, Lazzarotto T, Alain S (2017). Congenital cytomegalovirus infection in pregnancy and the neonate: consensus recommendations for prevention, diagnosis, and therapy. Lancet Infect Dis.

[7] Dollard S, Grosse S, Ross D (2007). New estimates of the prevalence of neurological and sensory sequelae and mortality associated with congenital cytomegalovirus infection. Rev Med Virol.

[8] Goderis J, De Leenheer E, Smets K, Van Hoecke H, Keymeulen A, Dhooge I (2014). Hearing loss and congenital CMV infection: a systematic review [Internet]. Pediatrics.

[9] Zagólski O (2008). Vestibular-evoked myogenic potentials and caloric stimulation in infants with congenital cytomegalovirus infection. J Laryngol Otol.

[10] Teissier N, Bernard S, Quesnel S, Van Den Abbeele T (2016). Audiovestibular consequences of congenital cytomegalovirus infection [Internet]. Eur Ann Otorhinolaryngol Head Neck Dis.

[11] Maes L, De Kegel A, Van Waelvelde H, De Leenheer E, Van Hoecke H, Goderis J (2017). Comparison of the motor performance and vestibular function in infants with a congenital cytomegalovirus infection or a Connexin 26 mutation: a preliminary study. Ear Hear.

[12] Pinninti S, Christy J, Almutairi A, Cochrane G, Fowler KB, Boppana S (2021). Vestibular, gaze, and balance disorders in asymptomatic congenital cytomegalovirus infection. Pediatrics.

[13] Goderis J, De Leenheer E, Smets K, Van Hoecke H, Keymeulen A, Dhooge I (2014). Hearing loss and congenital CMV infection: a systematic review. Pediatrics.

[14] Singh A, Heet H, Guggenheim DS, Lim M, Garg B, Bao M (2022). A systematic review on the association between vestibular dysfunction and balance performance in children with hearing loss. Ear Hear.

[15] Lieu JEC, Kenna M, Anne S, Davidson L (2020). Hearing loss in children: a review. JAMA.

[16] Lazzarotto T, Blázquez-Gamero D, Delforge ML, Foulon I, Luck S, Modrow S (2020). Congenital cytomegalovirus infection: a narrative review of the issues in screening and management from a panel of European experts. Front Pediatr.

[17] Puhakka L, Lappalainen M, Lönnqvist T, Niemensivu R, Lindahl P, Nieminen T (2019). The burden of congenital cytomegalovirus infection: a prospective cohort study of 20 000 infants in Finland. J Pediatric Infect Dis Soc.

[18] Boppana S, Ross S, Shimamura M, Palmer A, Ahmed A, Michaels M (2011). Saliva polymerase-chain-reaction assay for cytomegalovirus screening in newborns. N Engl J Med.

[19] Puhakka L, Lappalainen M, Lönnqvist T, Nieminen T, Boppana S, Saxen H (2022). Hearing outcome in congenitally CMV infected children in Finland—results from follow-up after three years age. Int J Pediatr Otorhinolaryngol.

[20] Janky KL, Rodriguez AI (2018). Contemporary concepts in pediatric vestibular assessment and management: quantitative vestibular function testing in the pediatric population. Semin Hear.

[21] Ross LM, Helminski JO (2016). Test-retest and interrater reliability of the video head impulse test in the pediatric population. Otol Neurotol.

[22] Bernard S, Wiener-Vacher S, Van Den Abbeele T, Teissier N (2015). Vestibular disorders in children with congenital cytomegalovirus infection. Pediatrics.

[23] Dhondt C, Maes L, Rombaut L, Martens S, Vanaudenaerde S, Van Hoecke H (2020). Vestibular function in children with a congenital cytomegalovirus infection: 3 years of follow-up. Ear Hear.

[24] Dhondt C, Maes L, Oostra A, Dhooge I (2019). Episodic vestibular symptoms in children with a congenital cytomegalovirus infection: a case series. Otol Neurotol.

